# Long-Term Outcome in a Phase II Study of Regional Hyperthermia Added to Preoperative Radiochemotherapy in Locally Advanced and Recurrent Rectal Adenocarcinomas

**DOI:** 10.3390/cancers14030705

**Published:** 2022-01-29

**Authors:** Baard-Christian Schem, Frank Pfeffer, Martin Anton Ott, Johan N. Wiig, Nils Sletteskog, Torbjørn Frøystein, Mette Pernille Myklebust, Sabine Leh, Olav Dahl, Olav Mella

**Affiliations:** 1 The Western Norway Regional Health Authority, 4034 Stavanger, Norway; trazom@online.no; 2 Department of Gastrointestinal Surgery, Haukeland University Hospital and Institute of Clinical Medicine, Medical Faculty, University of Bergen, 5021 Bergen, Norway; frank.pfeffer@uib.no; 3 Department of Surgery, Haugesund Hospital, Helse Fonna, 5504 Haugesund, Norway; martin.anton.ott@helse-fonna.no; 4 Department of Gastroenterological Surgery, Section of Surgical Oncology, Norwegian Radium Hospital, Oslo University Hospital HF, 0424 Oslo, Norway; joniwii@online.no; 5 Department of Surgery, Førde Central Hospital, 6807 Førde, Norway; nils.sletteskog@helse-forde.no; 6 Department of Oncology and Medical Physics, Haukeland University Hospital, 5021 Bergen, Norway; torbjorn.froystein@helse-bergen.no (T.F.); mette.pernille.myklebust@helse-bergen.no (M.P.M.); olav.mella@helse-bergen.no (O.M.); 7 Department of Pathology, Haukeland University Hospital and Institute of Clinical Medicine, Medical Faculty, University of Bergen, 5021 Bergen, Norway; sabine.leh@helse-bergen.no; 8 Medical Faculty, Institute of Clinical Science, University of Bergen, 5021 Bergen, Norway

**Keywords:** rectal cancer, hyperthermia, chemoradiotherapy, tumour control

## Abstract

**Simple Summary:**

Regional hyperthermia added to standard preoperative chemoradiotherapy for locally advanced and recurrent rectal cancer gives a high complete response rate and an improved long-term recurrence free survival.

**Abstract:**

Hyperthermia was added to standard preoperative chemoradiation for rectal adenocarcinomas in a phase II study. Patients with T3-4 N0-2 M0 rectal cancer or local recurrences were included. Radiation dose was 54 Gy combined with capecitabine 825 mg/m^2^ × 2 daily and once weekly oxaliplatin 55 mg/m^2^. Regional hyperthermia aimed at 41.5–42.5 °C for 60 min combined with oxaliplatin infusion. Radical surgery with total or extended TME technique, was scheduled at 6–8 weeks after radiation. From April 2003 to April 2008, a total of 49 eligible patients were recruited. Median number of hyperthermia sessions were 5.4. A total of 47 out of 49 patients (96%) had the scheduled surgery, which was clinically radical in 44 patients. Complete tumour regression occurred in 29.8% of the patients who also exhibited statistically significantly better RFS and CSS. Rate of local recurrence alone at 10 years was 9.1%, distant metastases alone occurred in 25.6%, including local recurrences 40.4%. RFS for all patients was 54.8% after 5 years and CSS was 73.5%. Patients with T50 temperatures in tumours above median 39.9 °C had better RFS, 66.7% vs. 31.3%, *p* = 0.047, indicating a role of hyperthermia. Toxicity was acceptable.

## 1. Introduction

Patients presenting locally advanced rectal cancer (LARC) or primarily non-resectable rectal cancer have a dire prognosis [1]. Locally recurrent rectal cancer is difficult to control [2,3]. Preoperative radiation for advanced rectal cancer [4,5,6] and palliative radiotherapy for metastasized or irresectable rectal cancer [7,8,9] is well established. 

The clinical benefit of superficial hyperthermia for malignant melanomas and deep pelvic hyperthermia as an adjuvant to radiotherapy for cervical cancer has been documented [10,11,12,13]. Additive effect of some chemotherapeutic drugs, including cisplatin have been shown experimentally [14]. It was therefore of interest to explore whether a preoperative combination of radiation, chemotherapy and hyperthermia could improve the results of surgery for rectal cancer. At the time the study was initiated, few clinical studies had treated primary rectal cancer by radiation combined with deep hyperthermia [13,15,16,17]. The combination of radiation with capecitabine was also new [18], as were the combination of hyperthermia and oxaliplatin in rectal recurrences [19] and experimental cell cultures [20,21]. Whole body hyperthermia and oxaliplatin in colorectal cancer patients was an experimental option not in general use [22]. We therefore first performed a phase I study to assess the feasibility of a combination of radiation, 5-day continuously administered 5-fluorouracil (5-FU), and weekly hyperthermia and oxaliplatin before surgery for rectal cancer [23]. Excellent local control was achieved, but acute diarrhoea was a frequent side effect. As the continuous administration of 5-FU via a central venous line was cumbersome in an outpatient setting, we replaced 5-FU with oral capecitabine in the current new phase II study. As various heating techniques had been used in earlier studies [17,24], we decided to explore the use of regional hyperthermia following the European quality assurance guidelines [25]. During the follow up of our patients, promising results have been published for LARC patients [26,27].

The present phase II study was open for patients with LARC without distant metastases and patients with local recurrences considered to be surgically curable, some with sufficient tumour shrinkage after preoperative treatment. During and after the inclusion and follow up of the study, the standard treatment has changed regarding radiation dose and inclusion of chemotherapy. We therefore waited with publication until long-term results were available. The present series of advanced and recurrent rectal cancer patients with good local control warrant consideration among the new therapeutic options. The secondary aim is the presentation of treatment-related toxicity.

## 2. Materials and Methods

From April 2003 to April 2008, 50 patients with histologically verified rectal adenocarcinomas were recruited. By a mistake, one patient with locally advanced tumour was examined with abdominal computed tomography (CT) the day after first treatment, which revealed multiple liver metastases, and this patient was therefore excluded due to major protocol violation.

### 2.1. The Inclusion Criteria

The inclusion criteria were patients with LARC (defined as T3 and T4 tumours with distance to the mesorectal facia less than 3 mm on magnetic resonance imaging (MRI), within 15 cm from the anal verge by proctoscopy) or recurrence after surgery alone. Age was below 76 years. The patients should not have evidence of distant metastases, and performance status should be 0–2. The patients should not have hypertension, cardiac failure or myocardial infarction in the previous 6 months. No chronic pulmonary or renal disease. No prior radiation or cancers, except basal cell carcinomas or stage 0 cervical cancer. Haematological tests should demonstrate Hgb > 10 g/dL, leucocytes > 3 × 10^9^/L or thrombocytes > 100 × 10^9^/L, and creatinine clearance > 30 mL/min. Due to the hyperthermia applicator size, maximal pelvic diameter should be less than 49 cm, and the patient should have no pacemaker or other metallic implanted object. Diagnostic procedures included clinical examination, rigid rectoscopy by a senior surgeon, biopsy confirming adenocarcinoma, CT of the pelvic area and abdomen with contrast in the rectum, and MRI of the pelvic area. Endorectal ultrasound was optional, and cystoscopy was performed if any invasion of the bladder was suspected. Blood counts included Hgb, leucocytes with differential counts, thrombocytes, analysis of Na, K, Cl, Ca, Mg and creatinine, bilirubin, ASAT, ALAT, ϒ-GT, LDH and creatinine kinase.

The primary tumours were classified according to TNM 4th edition [28]. Indications for preoperative radiotherapy of LARC was based on the MRI examination showing a threatened circumferential resection margin with less than 3 mm from the mesorectal fascia (MRF) according to Norwegian national guidelines, or tumour deposits outside the MRF. 

### 2.2. Statistics and Ethics

The outcomes were defined as local control, relapse-free survival included any local or distant recurrence with secondary colorectal cancer censored (RFS), cancer recti-specific survival (CSS) and overall survival (OS) as defined by Punt [29]. Toxicity was graded according to Common Terminology Criteria for Adverse Events v3.0 (CTCAE), Publish Date: 9 August 2003 (https://ctep.cancer.gov/protocoldevelopment/electronic_applications/docs/ctcaev3.pdf, accessed on 8 September 2020) [30].

Survival was assessed by Kaplan-Meier estimates with 95% confidence intervals (95% CI), and differences assessed by the log-rank test. Differences with a two-tailed *p*-value less than 0.05 were considered statistically significant. IBM SPSS version 26 (IBM Corp, Armonk, NY, USA) and R (version 4.0.3, R Foundation for Statistical Computing, Vienna, Austria) were used for analyses and creating survival curves.

After oral and written information on the experimental nature of the study, all patients signed a written informed consent form. The study was conducted according to the guidelines of the Declaration of Helsinki. It was approved by the Regional Ethics Committee, Health Region West (REK III nr. 159.01) and thereby in accordance with Norwegian law and regulations.

## 3. Treatment

Radiation therapy was based on 3D dose planning, mostly a three-field technique with one backfield and two side fields. Tumour with regional glands received 2 Gy × 23 with a boost of 2 Gy × 4–5 against primary tumour and mesorectum or metastatic lymph nodes. Total tumour dose was therefore 54–56 Gy based on tumour size and bowel volume to be included in the boost volume, as well as comorbidity, age and acute side effects. The treatment was administered once daily, 5 days weekly. Pauses except Saturdays and Sundays were compensated for by a 6th fraction the following week(s).

**Figure 1:**
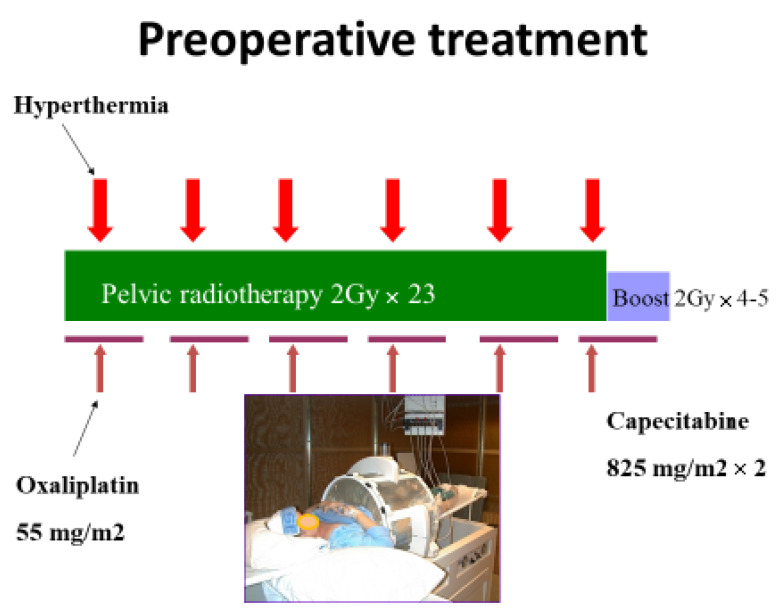
Treatment schedule for preoperative combination treatment by radiation, chemotherapy and hyperthermia for rectal adenocarcinoma.

Chemotherapy was administered as peroral capecitabine 825 mg/m^2^ 5 days a week, concomitant with radiation (See Figure 1). Maximal dose was 1650 mg/day, administered in one dose the evening before radiation and the other half in the morning before radiation. Oxaliplatin was administered at a dose of 55 mg/m^2^ each week, with a maximal dose 100 mg. Oxaliplatin was given as infusion during the hyperthermia session; at least five infusions, and if possible six infusions were administered. The drug was infused in 5% glucose, administered as a 2 h peripheral vein infusion, which started 1 h before planned start of hyperthermia.

Regional hyperthermia was administered by a BSD 2000 machine (Pyrexar Medical, Salt Lake City, UT, USA), using the Sigma-Eye applicator or the Sigma-60 applicator for patients with the highest pelvic diameters [17]. Hyperthermia was administered once a week from the first or second day of radiation, each treatment given as soon as possible after receiving the radiation fraction. Prior to radiation, catheters for Bowman temperature probes were inserted into tumour tissues using local anesthetics, and in women also in the vagina. Usually, a single catheter was inserted into the tumour, but for large tumours two catheters were used to optimize monitoring of the tumour heating. With problems of insertion of catheters or shrinkage of tumours, the probe measured rectal luminal temperature. One catheter was inserted in the urinary bladder. The positions of the catheters were verified by CT before the radiation. The treatment time was 60 min, calculated from the time the temperature probe in the tumour (or in rectal lumen if no probe was inserted in the tumour) reached 41.0 °C or started 30 min after initiation of the hyperthermia session. Normal tissues should be kept below 43.0 °C. The focus of heating was controlled by phase and amplitude steering [31]. If the awake patient reported discomfort or pain repeated doses of fentanyl up to 0.10 mg was given intravenously. With continuous bladder temperatures > 42.5 °C, the bladder was irrigated by 10–30 mL of isotone saline holding room temperature for cooling. Bladder installations were kept at a minimum to avoid cooling of possible tumour near the bladder. Only tumour-tissue temperature-probe data are used for quality assessment of the hyperthermia given. The T20, T50 and T90 are defined as the temperature equal to or exceeded by 20%, 50% and 90% of the measured temperatures, respectively [32], and were calculated using the RhyThM software [33]. 

Surgery. Rectal resections were performed according to total mesorectal excision (TME) principles. A partial, total or extended TME was carried out depending on the location. With invasion of neighbouring organs in locally advanced or recurrent tumours, a total pelvic exenteration procedures was performed. Totally 25 patients had a permanent colostomy. The resection margins were classified by the pathologists as R0 resection with a margin > 1 mm, R1 with a margin < 1 mm, and R2 in case of involved margins. The tumour regression grade (TRG) was classified by the Dworak criteria [34]. Follow up after surgery for at least 5 years followed the Norwegian national guidelines.

## 4. Results

In total 43 patients with LARC and 6 patients with recurrent rectal tumours without previous preoperative radio (chemo)therapy, 32 male and 17 female, were included. The characteristics for the patients are shown in Table 1. Fourteen tumours were locally advanced rectal adenocarcinomas, with growth into or beyond the MRF (two patients with T3N1 tumours had only 3 and 2 mm to the MRF) and were considered moderate-risk patients, while 29 patients had high-risk tumours.

The median radiation dose was 54.0 Gy (range 50–56, of these, seven received 56 Gy). The median number of hyperthermia sessions was 5.4 (range 1–6) and 92% of the patients had at least four hyperthermia sessions. The temperature data measured in the tumours (*n* = 152 catheters) was mean (T-mean) 39.92 °C (95%CI 38.22–41.62), T-min 39.14 °C (95%CI 37.6–40.68), T-max 40.61 °C (95%CI 39.56–41.66), T20 was 40.29 °C (39.32–41.26), T50 39.91 °C (95%CI 39.06–40.76), and T90 was 39.36 °C (95%CI 38.58–40.14). There were no significant relation between T-stage, primary tumours versus recurrences or T size and median T50.

All patients had at least one oxaliplatin dose, 94% had four and more doses, and 71% had all six scheduled courses. One patient had only three weeks with capecitabine, two only four weeks, while 94% had the scheduled 5–6 weeks of oral chemotherapy together with radiation.

A total of 41 of the 43 patients with locally advanced tumours had the scheduled surgery at a median time of 84 days (range 69–216) after the start of radiation. The longest delay was for a very advanced tumour, which first had an exploratory laparotomy finding that the tumour was irresectable, and a new attempt later with the successful removal of a seemingly large tumour, however showing no vital tumour cells when examined by microscopy. Two patients were not operated as planned preoperative examinations revealed distant metastases. All six patients with local recurrences were operated as scheduled. The resections were recorded as R0 in 41 (87%) of the operated patients, R1 in 3 (6%) and R2 in 3 (6%) patients. The pathological assessment of TRG showed no malignant cells, complete regression (TRG4, pCR) in 14 (29.8%) of the specimens, 22 (46.9%) with TRG 3, 7 (14.9%) with moderate response, TRG 2, and only 4 (8.5%) with minimal regression, TRG 1.

**Figure 2:**
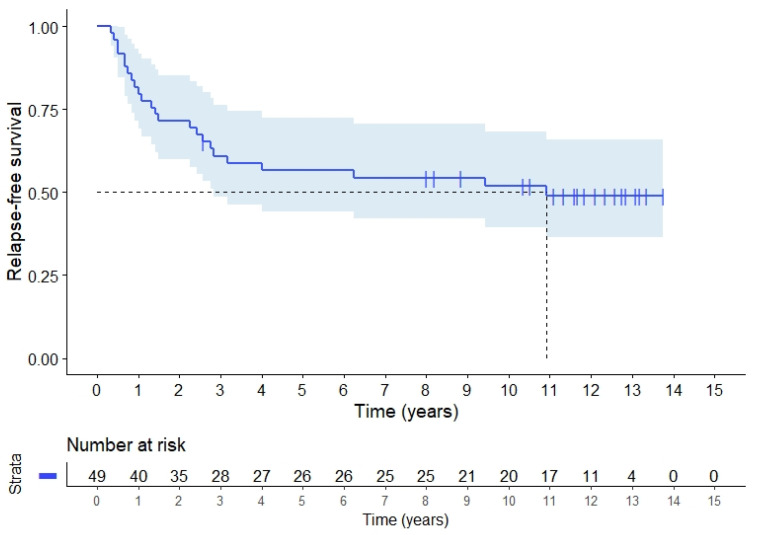
Relapse-free survival (RFS) for 49 rectal cancer patients with locally advanced or recurrent rectal adenocarcinoma.

**Figure 3:**
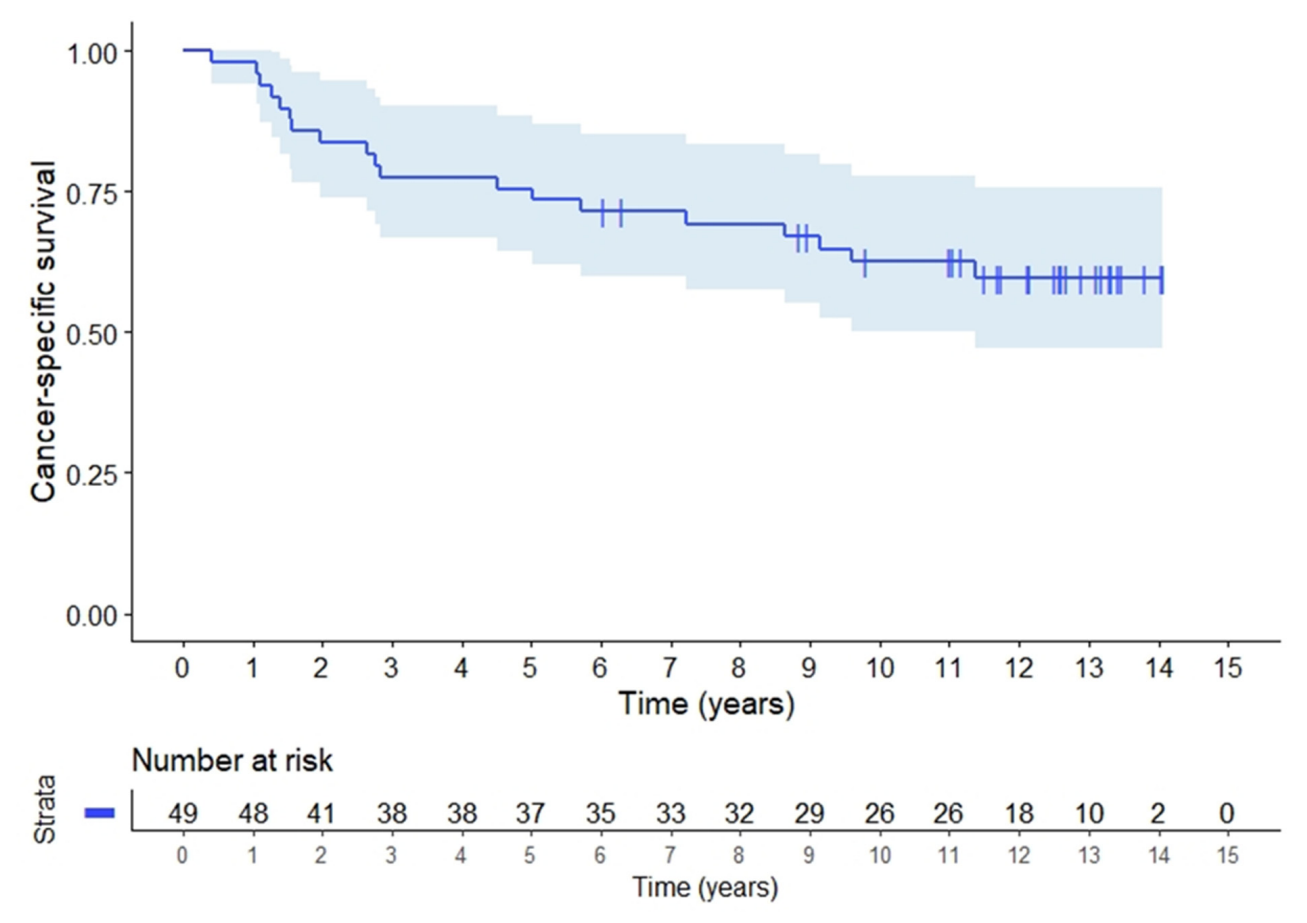
Cancer specific survival (CSS) for 49 patients, 43 with locally advanced and 6 patients with local recurrences of rectal adenocarcinomas.

**Figure A1:**
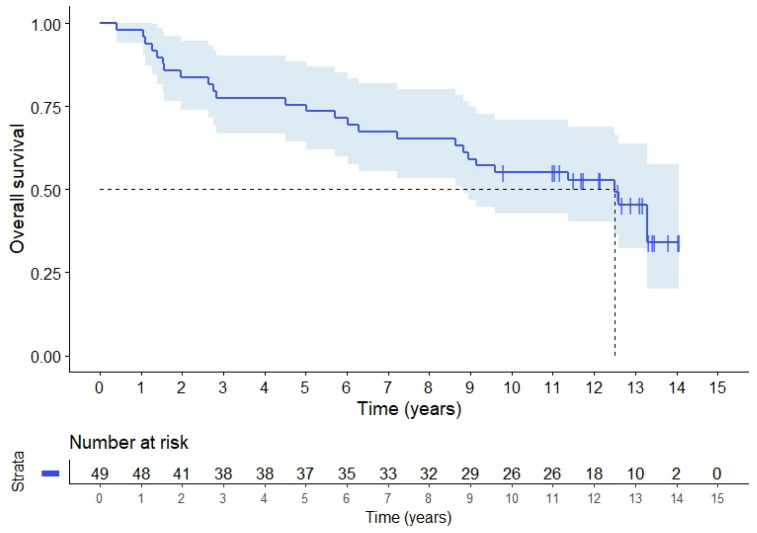
Overall survival for 49 rectal cancer patients, 43 with locally advanced and 6 with recurrent adenocarcinomas.

The rate of local recurrence alone at 5 and 10 years was 9.1% (95%CI 4.4–13.8) and at 15 years the local recurrence rate was 12.3% (95%CI 0.8–23.8) for all included patients. Distant metastases only occurred in 25.6% (95%CI 12.3–38.9) of the patients after 5 years, 40.4% (95%CI 26.4–54.4) including concurrent local recurrences. For all patients, 5-year RFS was 54.8% (95%CI 40.8–68.8), Figure 2. There was no difference in RFS according to presentation as locally advanced tumours or recurrences. CSS for all patients at 5 years was 73.5% (95%CI 61.2–85.8) and at 10 years 62.5% (95%CI 48.9–76.2), Figure 3. CSS was similar for LARC patients and patients with recurrence. OS was 73.5% (95%CI 61.2–85.8) at 5 years, and dropped to 55.1% (95%CI 41.3–68.9) after 10 years (Figure A1).

**Figure 4:**
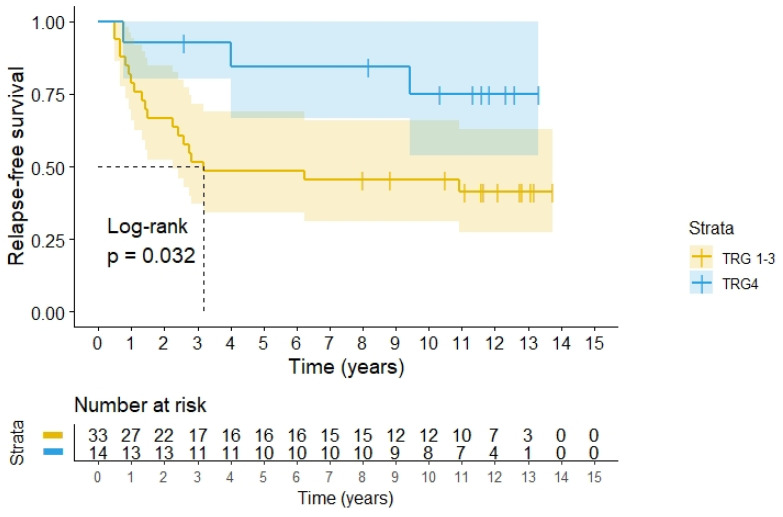
RFS according to histopathology classification according to Dworak’s TRG grades for 47 patients operated for rectal adenocarcinomas after preoperative radiochemotherapy with hyperthermia. TRG4 means pCR.

Classification of tumour response as TRG 4 (pCR) among the 47 operated patients yielded better RFS compared with the other groups (*p* = 0.032), see Figure 4. For patients with TRG 4 the 5-year CSS was 92.9% (95%CI 79.4–100.0) versus 72.7% (95%CI 57.5–87.9) for patients with residual tumour cells, and 10-year CSS 83.6 % (95%CI 62.5–100) versus 57.4% (95%CI 40.0–74.8), (*p* = 0.063), respectively. Thus the CSS differences were not statistically significant.

**Figure 5:**
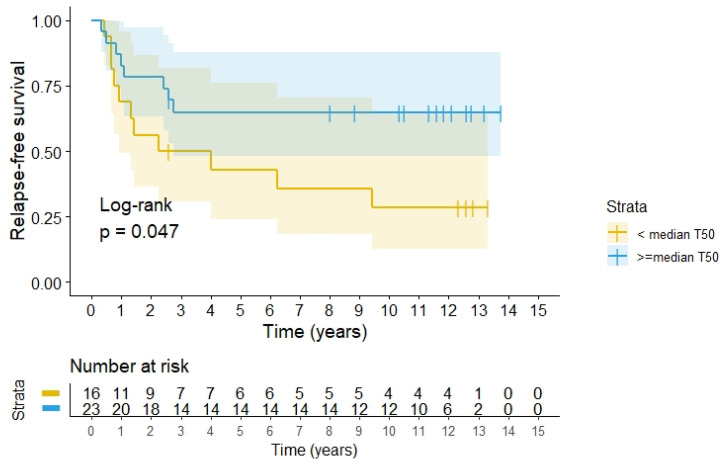
RFS according to quality of heating divided above or below 39.91 °C, the median of the T50 measured in the tumours during all heating sessions among 39 patients.

When evaluating outcome in relation to the thermometry data, we were only able to retrieve the original disk recordings for 39 patients, thus data were unavailable for 10 patients. It was found that the RFS was significantly better, 66.7% (95%CI 40.6–86.7) for patients with recordings above 39.9 °C (T50, median of all recordings) versus 31.3% (95%CI 9.3–53.3), *p* = 0.047, in the lower group, see Figure 5. 

Toxicity. Table 2 shows that most patients who had the scheduled treatment including surgery, had some acute side effects: 23% had grade 1, 34% had grade 2, 40% had grade 3, and only one (2%) recorded as grade 4 due to reduced general condition caused by several side effects. The most frequent side effects were diarrhoea due to chemotherapy and radiation, skin toxicity due to radiation and nausea related to chemotherapy. Fever reaction without infection after oxaliplatin was recorded in 40% of the patients. Twenty-seven patients (57%) experienced no long-term toxicity. Grade 1 occurred in two patients (4%, subileus and an accidentally fixed urether catheter), grade 2 in four patients (8%, one slight vaginal athresia and one abdominal pain possibly related to the oncological treatment, one patient had surgery for a ventral hernia, and one patient was not operated on due to liver metastases presented a Guillain Barre syndrome, probably unrelated to the treatment). Grade 3 was recorded in six patients (12%), two patients with bladder symptoms (irritation and paresis), one hip arthrosis and one subileus after several years, and one stoma surgery due to local pain. Grade 4 was observed in five patients (10%): two with stenotic ureters, two patients had ileus, one of them also hip surgery and one pelvic pain. One Grade 5 patient died 2 weeks after surgery due to sepsis and adult respiratory distress syndrome, considered a complication after major surgery. In summary the side effects were as expected for the present cohort of locally advanced tumours with some elderly patients, and no obvious unexpected toxicity related to the hyperthermia treatment was observed.

## 5. Discussion

Since this study was done, the surgical techniques used in combination with radiation and delivery of radiation have evolved and have reduced the local recurrence rate to about 5% for operable rectal cancer [35,36]. We therefore were initially not too enthusiastic of our achievement with the use of hyperthermia. However, most of the patients could be resected 12 weeks after the preoperative treatment in our series. We obtained a R0 resection rate of 87%, which seems better than 61% in a previous national study including locally advanced T4 rectal cancer patients [37]. Pathological assessment of the operation specimens revealed no residual tumour cells (TRG 4) in 29.8% of the patients, which is higher than the standard 10–20% pCR as reported after preoperative radiochemotherapy for rectal cancer [38,39,40,41,42], but some report even higher percentages [39,43,44]. In general the TRG4 rate is influenced by the initial tumour stage, the given treatment, as well as the timing between preoperative treatment and surgery. The high TRG4 rate in the present cohort of advanced rectal cancers is very promising. The most important aspect of the TRG4 is that no viable tumour cells after preoperative treatment is a predictor of higher RFS as demonstrated in Figure 4 in accordance with the findings in other series [40,45,46,47,48]. In the pCR group, 11 of 14 patients had no recurrence versus 14 of 33 in the other categories (*p* < 0.05). In our cohort the 5-year CSS was 92.9% for patients with pCR versus 72.7% with residual tumour cells, but this did not reach statistical significance, possibly due to the small sample size.

The isolated local recurrence rate at 5 years was 9% in this series of locally advanced rectal cancer patients. This is the same as observed in 2005 in a series of 3388 Norwegian patients with operable rectal cancer treated for cure by surgery alone including 5% preoperative and 5% postoperative radiation [48]. In a previous Norwegian series of LARC patients defined as T4 tumours, the local recurrence rates at 5 years were 18% (95%CI 14–23) for patients with a R0-resection and 40% (95%CI 26–52) if the procedure was classified as R1 [35]. We observed two local recurrences after about 10 years. Whether these recurrences are true local recurrences or de novo development of new primary tumours cannot be determined, and we have therefore recorded them as local recurrences. Habr-Gama has reported adenomas after pCR for rectal cancer [49]. Thus, new primary cancer at the tumour bed may develop from such residual adenomas.

For comparison, the 5-year relative survival rate for all operated rectal cancers in Norway was 79% in 2004-06 [35]. The overall survival at 5 years in our series was 73.5%, clearly improved from 29% in the previous Norwegian series where 5-year survival was 49% after R0 resection and 20% after a R1 resection. In a more recent randomized trial, the 5-year overall survival for locally advanced rectal cancer was 66% after preoperative radiochemotherapy and 53% after preoperative radiation alone [1]. However, after 10-years there was no significant difference between radiation alone or radiochemotherapy in this trial [49].

The current study was designed to assess a possible role of hyperthermia. When 86 rectal cancer patients, treated with preoperative radiotherapy followed by surgical resection and adjuvant 5-FU and hyperthermia once or twice a week, were compared with predicted outcomes from a nomogram based on randomised European trials without hyperthermia, the observed OS (87.3%) versus predicted OS (75.5%), distant metastasis free survival (87.3%) vs. predicted (75.5%), and local control (95.8% vs. 95.8%, respectively), was better after hyperthermia [50]. Recently a series of 112 patients with locally advanced and recurrent rectal cancer had preoperative radiation (55.8–59.4 Gy) and regional hyperthermia with 5FU or capecitabine and oxaliplatin in locally advanced patients [51]. They reported a local recurrence rate of 2.3% with hyperthermia vs. 21.3% without hyperthermia and DFS of 89.1% vs. 70.4%, respectively. In another study including 78 LARC patients also using a similar regimen as our study with radiation 50.4 Gy combined with 5-FU and hyperthermia twice per week, pCR was seen in 14% with combined TRG 4 and TRG 3 in 50% of the patients [52]. DFS and OS were also in accord with our data. They also report that those patients achieving best quality hyperthermia had best tumour response. These studies therefore also support a role of hyperthermia in rectal cancer. Currently there has not been published a prospectively randomized study demonstrating the effect of deep hyperthermia added to preoperative treatment in rectal cancer patients.

It proved difficult to achieve the temperature aim stated in our protocol in most patients, mainly due to local pain and discomfort limiting power output, despite the use of fentanyl. However, achieving a temperature above median of T50 for the patients where temperature was recorded in tumours, resulted in a significant better RFS. Our findings support general reviews demonstrating that hyperthermia in addition to its own effect, is a sensitizer for radiation [27,53,54,55] as well as a chemosensitizer [53]. For further details on mechanism of action of hyperthermia, see references [12,53,56]. In addition, hyperthermia may also have positive immunological effects [57,58].

The role of oxaliplatin in the preoperative treatment of rectal cancer is controversial. Some authors report only increased toxicity (diarrhoea) [59], while others showed improved pCR, local control and distant metastases, but no effect on OS [60]. Other authors have reported improved outcomes in patients treated with oxaliplatin [61,62,63]. Avoiding the last scheduled dose of oxaliplatin significantly reduced pathological response in a recent study [64]. We cannot assess the contribution of oxaliplatin in our cohort, but we notice that oxaliplatin is part of current total neoadjuvant therapy for rectal cancer where induction chemotherapy is used before radiation [65,66,67,68]. Giving more chemotherapy before surgery was recently documented in two large randomized studies and several phase II studies, yielding pCR rates around 30% for total neoadjuvant therapy versus 14% for standard radiochemotherapy in a meta-analysis [69,70,71]. Although the disease free survival (DFS) at 3 years seems promising [71], the 4.6 year local recurrence rate was 8.3% in the experimental arm versus 6% after standard radiochemotherapy in the Rapido trial [69]. It should be noted that the time from diagnosis to surgery was 10 weeks longer in the experimental arm, which may have favoured this group. Distant metastases were recorded for 20.0% in the experimental group compared with 26.8% in the standard group in this trial, and disease-related treatment failure was 23.7% in the experimental group versus 30.4% (*p* = 0.019) in the standard of care group, but there was no difference in OS. Therefore, the effect of this more intense preoperative chemotherapy treatment on reduction of distant metastases must be further validation in new studies to assess the impact of more intense chemotherapy over longer time. Preoperative chemotherapy with mFOLFIRINOX may be an alternative option as pCR was 27.5% after chemotherapy alone versus 11.7% after standard preoperative chemoradiation followed by adjuvant chemotherapy, and 3-year DFS was 75.7% and 68.5%, respectively [72].

Surgery alone or after preoperative radiation has been the traditional treatment of local recurrences of rectal cancer [73,74]. Radiotherapy alone yields poor local control after surgery for rectal cancer [2], while radiochemotherapy followed by surgery seems better [49]. Currently carbon-ion radiotherapy seems to offer even better local control, but distant failures remain a problem [75,76]. Our data and the recent phase II studies imply a role of hyperthermia in recurrent rectal cancer [51,52].

In the present study, there was no unexpected long-term toxicity associated with addition of hyperthermia and the overall acute and long-term toxicity seems to be in line with current use of preoperative radiochemotherapy for rectal cancer [1,77,78]. No negative effects on quality of life with addition of hyperthermia to neoadjuvant radiochemotherapy was reported in this setting [79]. We must however admit that side effects recorded in the patient’s journal and retrospectively collected as done in our study, are a weakness of our study.

## 6. Conclusions

This study of radiochemotherapy combined with deep regional hyperthermia showed, after long follow-up, good local control and survival, and no indications of increased treatment-related long-term side effects. The achieved temperatures during hyperthermia were relatively low, indicating a possibility for even better tumour effects with improvement of heating technology.

## Figures and Tables

**Table 1:** Patient characteristics for patients included and treated according to the study protocol.

Category	Total	Male	Female
Included	49	32	17
Age (years)	59.1 (range 21.0–75.6)	60.3 (range 21.0–75.6)	56.9 (range 39.4–74.7)
**Locally advanced**	43	27	16
Level 0–5 cm	19	15	4
Level 6–10 cm	21	11	10
Level 11–15 cm	3	1	2
Mean height cm	6.7	6.1	7.4
T3	19	11	8
T4	24	16	8
N0	3	3	0
N1	17	11	1
N2	21	12	9
Nx	2	1	1
Moderate	14	7	7
High risk	29	20	9
**Local recurrence**	6	5	1

**Table 2:** Acute toxicity observed during preoperative therapy and first month after surgery in 47 patients.

Grade	Maximal Acute Toxicity	Skin Toxicity	Diarrhoea	General Condition	Urinary	Nausea	Oxaliplatin Fever	Other
1	11	5	17	2	4	8	11	5
2	16	4	13	7	3	4	1	9
3	19	3	7	3	0	0	2	9
4	1	0	0	1	0	0	0	0
No surgery	2							

## Data Availability

Anonymous data may be disclosed upon any reasonable relevant request according to EU General Data Protection Regulation (GDPR) and Norwegian legislation.
